# Treatment outcomes and healthcare resource utilization in critically ill COVID-19 patients in Korea: A nationwide multicenter cohort study

**DOI:** 10.1097/MD.0000000000040505

**Published:** 2024-11-15

**Authors:** Taehwa Kim, Jeong Su Kim, Min Wook So, Hye Ju Yeo, Jin Ho Jang, Onyu Park, Woo Hyun Cho

**Affiliations:** a Department of Internal Medicine, Pusan National University School of Medicine, Busan, Republic of Korea; b Division of Allergy, Pulmonary, and Critical Care Medicine, Department of Internal Medicine, Yangsan, Republic of Korea; c Transplant Research Center, Research Institute for Convergence of Biomedical Science and Technology, Pusan National University Yangsan Hospital, Yangsan, Republic of Korea; d Division of Rheumatology, Department of Internal Medicine, Pusan National University Yangsan Hospital, Pusan National University School of Medicine, Yangsan, Republic of Korea; e College of Nursing, Research Institute of Nursing Science, Pusan National University, Yangsan, Republic of Korea.

**Keywords:** COVID-19, critically ill COVID-19, national data, pneumonia

## Abstract

COVID-19 pandemic was accompanied by many healthcare-related issues. Concrete national data regarding the care performance of critical ill cases of COVID-19 does not exist in Korea. The current study aimed to describe the treatment outcome and healthcare resource utilization of critically ill COVID-19 patients. Our multicenter retrospective cohort study enrolled critically ill COVID-19 patients from 22 tertiary care hospitals in Korea. Inclusion criteria: (1) patients aged 19 years or older, (2) patients with laboratory-confirmed SARS-CoV-2 infection who received at least one of following initial treatments such as high-flow oxygen therapy (HFOT) or noninvasive ventilation (NIV) or invasive mechanical ventilation (IMV) or extracorporeal membrane oxygenation. During the study wave, a total of 1358 eligible participants were enrolled, with 21 institutions participating in the study. Among them, data from 1113 patients were available and analyzed. Of 921 (82.7%), 621 (55.8%) were supported by IMV. Of the 921 patients supported by HFOT or NIV, 438 (47.6%) recovered without IMV, 429 (46.6%) required IMV, and 54 died who DNR after NIV was applied. Prone position ventilation was administered to 163 (33.1%) patients with IMV and 25 (6.2%) patients with HFOT. Extracorporeal membrane oxygenation was administered to 128 (20.6%) patients treated with IMV. The overall mortality rate was 26.4%. In South Korea, mortality rates for patients with severe COVID-19 pneumonia have been shown substantial fatality, with the highest mortality rates observed in wave 3. The increased mortality rate in wave 3 could be associated with the rapid escalation of critically ill COVID-19 patients and the consequent saturation of intensive care unit capacities. Patients received NIV therapy and prone position ventilation more frequently in wave 3 as the number of cases increased.

## 1. Introduction

The SARS-CoV-2 outbreak highlighted the challenges associated with limited healthcare resources during a surge of infectious spread and lack of policies addressing the equal or equitable distribution of resources.^[1–3]^ This directly impacted the health care system, including the critical care services, and the clinical outcomes of patients with severe COVID-19.^[4–7]^ In early 2020, there was no effective therapy against the viral infection. Quarantine and supportive treatment were the mainstays for patients hospitalized with severe SARS-COV-2 infection. The negative conversion of SARS-COV-2 RNA by RT-PCR was the only biomarker for release from quarantine for a considerable wave. The shortage of intensive care unit (ICU) resources almost reached the limit of locoregional collapse of healthcare system during the early pandemic wave.^[8]^ Healthcare policy makers in Korea declared the so-called 3T strategy (test, trace, and treatment)^[9]^ and tried to quarantine all PCR-confirmed cases at home or in dedicated hospitals.^[10]^ The strategy was effective in decreasing the surge of each variant’s spread compared to that in other countries.^[9]^ Despite the success of Korea in preventing an overwhelming number of case fatalities, its healthcare system was overloaded. This resulted in collateral damage to non-covid-critically ill patients, which is yet to be fully evaluated.^[11,12]^ Therefore, the pattern of healthcare resource use and outcome of critically ill COVID-19 patients deserved to be assessed. While there are many studies that have analyzed broader national trends and outcomes for COVID-19, there is no national compilation of ICU data. The current study may help designing policies that will guide the optimal utilization of healthcare resources through our accumulated experience and knowledge. This study aimed to collect data of critically ill COVID-19 patients and analyze the treatment patterns and outcomes of those who received critical care services during the early pandemic wave.

## 2. Methods

### 2.1. Study population

This was a retrospective multicenter cohort study of patients hospitalized with critically ill COVID-19 in 22 hospitals across Korea between January 1, 2020 and August 31, 2021. Patient inclusion criteria were the following: confirmed case of COVID-19, age 19 years or older, and hospitalization in an intensive care bed dedicated for patients with critically ill COVID-19. Critically ill COVID-19 was defined as requiring one of the following respiratory supports: 1. High-flow oxygen therapy (HFOT) or noninvasive ventilation (NIV), 2. Invasive mechanical ventilation, and 3. Extracorporeal membrane oxygenation (ECMO). The patient exclusion criteria were age <18 years, and mild or moderate disease that did not require respiratory support.

The average number of patients enrolled per the participating institutions during the study was 51, with the highest enrollment being 182 and the lowest being 5. For each wave, the average number of enrollments was 5 in wave 1, 7 in wave 2, and 41 in wave 3.

The study was approved by the institutional review boards of each hospital, including the Institutional Review Board of Pusan National University Yangsan Hospital (approval number: 04-2021-042, approved date: 07 October, 2021). The requirement for informed consent was waived, due to the retrospective nature of the design and a standard-of-care observational study with no intervention. The procedures were followed in accordance with the ethical standards on human experimentation by the responsible committee of each hospital and the Declaration of Helsinki, 1975.

### 2.2. Data collection

Critically ill COVID-19 patients admitted to a dedicated intensive care bed during the study wave were screened for eligibility by identifying the respiratory support devices used in their treatment. EMR-based medical records was collected from the time of admission till death or survival discharge from hospital. All participating hospitals used the same electronic case report form to collect data, which was then audited by the lead organization. The following information was collected: age, sex, demographic data, comorbidities, laboratory data, drugs administered, duration of ICU stay, duration of overall hospital stay, outcome data, clinical frailty scale, and coverage for mechanical ventilation and ECMO, treatment with prone position ventilation, and pharmacologic treatment and survivorship.

### 2.3. Data analyses

Patients were categorized into 2 groups (survival group and non-survival group) according to survivorship. Disease severity and clinical and laboratory variables of the 2 groups were compared and analyzed. Clinical factors associated with hospital mortality were evaluated using a Cox regression.

To describe the pattern of ICU resource usage according to each surge of infection, COVID-19 outbreak was classified by 3 time waves as follows: wave 1 (epidemic wave mainly in abroad cases and several regions), 20 January, 2020–11. August 2020; wave 2 (epidemic wave mainly in the metropolitan area), 12 August, 2020–12. November 2020, and wave 3 (epidemic wave mainly in the nationwide): 13 November, 2020–6. July 2021. The use of respiratory support and care and the clinical outcome were compared across the waves. The level of respiratory care and its outcome were detailed according to the time wave to describe the use of ICU resources according to severity.

### 2.4. Statistical analysis

Categorical and continuous variables were expressed as number (percentage) and mean ± standard deviation or median (interquartile range). They were compared using independent *t* test, and chi-square test. Multivariate analysis using Cox proportional hazards model with stepwise regression selection method was performed to identify the independent factors for hospital mortality. Kaplan–Meier curves with log-rank test were used for the analysis of hospital mortality. The Shapiro–Wilk test was used to assess the normality of continuous variables. For missing data with more than 5% missingness, we utilized the multiple imputation function in SPSS to handle missing values. All statistical analyses were performed using the MedCalc program version 22.009 and SPSS Statistics Software for Windows, Version 27.0. (IBM Corp., Armonk, NY).

## 3. Results

### 3.1. Baseline characteristics of COVID-19 patients based on survival data

**Figure 1.**
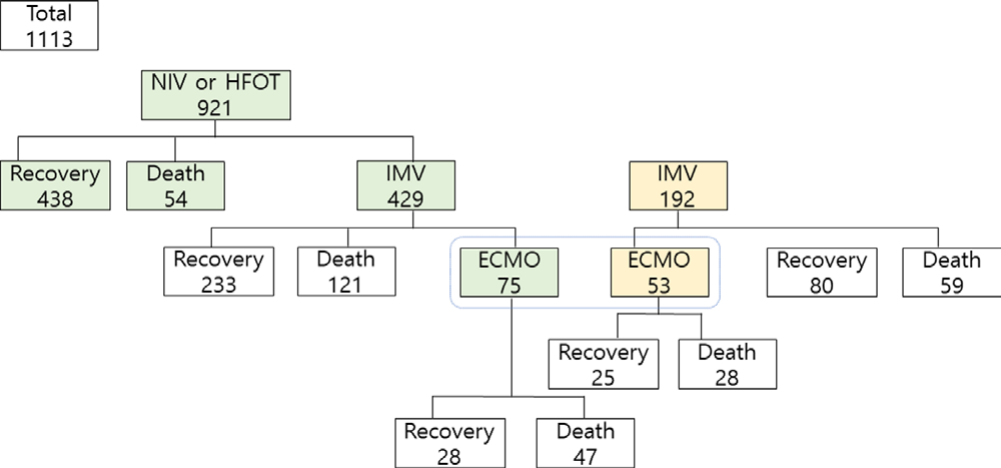
Study flow chart.

A total of 1358 patients were enrolled from the 21 participating hospitals in the study. Of those, patients with no final data were excluded, and 1113 patients were included in the analysis; 819 (73.6%) patients were discharged alive while 294 (26.4%) patients died (Fig. 1). Mean age of the patients was 67.6 ± 13.8 years, and approximately 60.6% of them were males. The mean body mass index was 24.7 ± 4.6 kg/m^2^, smoking habitus was 31.0%, and the most common comorbidity was hypertension. NIV or HFOT was used in 82.7% of cases, invasive mechanical ventilation was used in 55.8% of cases, and ECMO was used in 11.5% of cases.

The difference in clinical characteristics between survivors and non-survivors is presented in Table 1. Significant differences were observed between the survivor and non-survivor groups in terms of age, age group, height, weight, comorbidities, clinical frailty scales before admission, initial sequential organ failure assessment (SOFA), type of respiratory support, administration of remdesivir, and duration of ICU stay (survivor vs non-survivor group; age: 65.3 ± 13.6 vs 74.0 ± 12.4, *P* < .001; age group: *P* < .001; hypertension: 51.8% vs 59.2%, *P* = .029; coronary vascular disease: 9.9% vs 16.3%, *P* = .003; pulmonary disease: 6.3% vs 12.9%, *P* < .001; chronic neurological disease: 11.5% vs 20.1%, *P* < .001; chronic renal disease: 5.4% vs 12.2%, *P* < .001; solid malignant disease: 5.0% vs 13.3%, *P* < .001; clinical frailty scales before admission: 3.0 ± 1.6 vs 3.7 ± 2.0, *P* < .001; initial SOFA: 3.3 ± 2.0 vs 4.7 ± 2.7, *P* < .001; NIV or HFOT: 86.8% vs 71.4%, *P* < .001; invasive ventilation: 46.5% vs 81.6%, *P* < .001; ECMO: 6.8% vs 24.5%, *P* < .001; remdesivir: 75.7% vs 68.7%, *P* = .024; duration of ICU stay: 20.7 ± 23.2 vs 26.1 ± 23.3, *P* = .001).

**Table 1:** Baseline characteristics of patients with COVID-19 according to survival (N = 1113).

Characteristics	Survival (n = 819)	Non-survival (n = 294)	Overall (n = 1113)	*P*-value
Age (yrs)				
Mean	65.3 ± 13.6	74.0 ± 12.4	67.6 ± 13.8	<.001
19–29	24 (2.9)	3 (1.0)	27 (2.4)	<.001
30–49	137 (16.7)	19 (6.5)	156 (14.0)	
50–64	205 (25.0)	37 (12.6)	242 (21.7)	
65–79	327 (39.9)	130 (44.2)	457 (41.1)	
80–89	111 (13.6)	81 (27.6)	192 (17.3)	
≥90	15 (1.8)	24 (8.2)	39 (3.5)	
Sex				
Male	496 (60.6)	177 (60.2)	673 (60.5)	.914
Female	323 (39.4)	117 (39.8)	440 (39.5)	
BMI (kg/m^2^)	24.8 ± 4.5	24.3 ± 4.9	24.7 ± 4.6	.080
Smoking Hx.				
Yes	257 (31.4)	88 (29.9)	345 (31.0)	.645
No	562 (68.6)	206 (70.1)	768 (69.0)	
Comorbidities				
Hypertension	424 (51.8)	174 (59.2)	598 (53.7)	.029
Diabetes mellitus	267 (32.6)	109 (37.1)	376 (33.8)	.164
Coronary vascular disease	81 (9.9)	48 (16.3)	129 (11.6)	.003
Pulmonary disease	52 (6.3)	38 (12.9)	90 (8.1)	<.001
Chronic neurologic disease	94 (11.5)	59 (20.1)	153 (13.7)	<.001
Chronic Renal disease	44 (5.4)	36 (12.2)	80 (7.2)	<.001
Liver cirrhosis	20 (2.4)	10 (3.4)	30 (2.7)	.384
Immunocompromised state	17 (2.1)	9 (3.1)	26 (2.3)	.337
Connective tissue disease	16 (2.0)	2 (0.7)	18 (1.6)	.138
Hematology disease	9 (1.1)	6 (2.0)	15 (1.3)	.230
Solid malignant disease	41 (5.0)	39 (13.3)	80 (7.2)	<.001
Clinical frailty scale before admission	3.0 ± 1.6	3.7 ± 2.0	3.2 ± 1.8	<.001
Initial SOFA	3.3 ± 2.0	4.7 ± 2.7	3.7 ± 2.0	<.001
Respiratory support Type				
Noninvasive ventilation or HFOT (%)	711 (86.8)	210 (71.4)	921 (82.7)	<0.001
Invasive ventilation (%)	381 (46.5)	240 (81.6)	621 (55.8)	<0.001
ECMO (%)	56 (6.8)	72 (24.5)	128 (11.5)	<0.001
Drug treatments				
Remdesivir	618 (75.5)	202 (68.7)	820 (73.7)	0.024
Steroids	774 (94.5)	283 (96.3)	1057 (95.0)	0.238
Tocilizumab	75 (9.2)	20 (6.8)	95 (8.5)	0.385
Clinical outcome (day)				
Length of Hospital	33.3 ± 37.5	29.9 ± 24.3	32.4 ± 34.5	0.075
Length of ICU	20.7 ± 23.2	26.1 ± 23.3	22.1 ± 23.3	0.001

BMI = body mass index, ECMO = extracorporeal membrane oxygenation, HFOT = high-flow oxygen therapy, ICU = intensive care unit, SOFA = sequential organ failure assessment.

### 3.2. Overall outcome and types of respiratory and rescue therapies for COVID-19 patients according to each time wave

Upon comparison of the level of care across the time waves of COVID-19 surge, we found no difference in hospital mortality (wave 1 vs wave 2 vs 3 wave: 18.9% vs 23.0% vs 27.7%; *P* = .113). However, there were significant differences across the 3 time waves in terms of duration of hospital stay, duration of ICU stay, and ICU mortality. Regarding the baseline severity index, SOFA score was highest during wave 1 while no difference was observed in ratio of oxygen saturation index across the 3 groups (baseline SOFA, *P* = .035; ratio of oxygen saturation index, *P* = .485). For respiratory support, NIV or HFOT were administered more in waves 2 and 3 than in wave 1 and prone position ventilation was performed more in wave 2 in terms of time per session (hour/session) and total days of application (days) (Table 2).

**Table 2:** Overall outcome, types of respiratory and rescue therapies for patients with COVID-19 according to timeline.

Characteristics	COVID-19 infected patients by period			Overall (n = 1113)	*P*-value
	Period 1 Epidemic period mainly in abroad cases and several regions (20.1.20–8.11) (n = 95)	Period 2 Epidemic period mainly in the metropolitan area (20.8.12–11.12) (n = 135)	Period 3 Epidemic period mainly in the nationwide (20.11.13–21.7.6) (n = 883)		
Clinical outcome					
Length of Hospital	39.0 ± 38.8	39.5 ± 40.2	30.6 ± 32.8	32.4 ± 34.5	.002
Length of ICU	31.3 ± 38.3	25.9 ± 24.1	20.6 ± 20.6	22.1 ± 23.3	.007
Mortality of ICU	14 (14.7)	25 (18.5)	222 (25.1)	261 (23.5)	.002
Mortality of Hospital	18 (18.9)	31 (23.0)	245 (27.7)	294 (26.4)	.113
Respiratory support Type					
Noninvasive ventilation or HFOT (%)	70 (73.7)	106 (78.5)	745 (84.4)	921 (82.7)	.012
Invasive ventilation (%)	60 (63.2)	80 (59.3)	481 (54.5)	621 (55.8)	.185
ECMO (%)	13 (13.7)	15 (11.1)	100 (11.3)	128 (11.5)	.782
Severe index					
Baseline SOFA	4.1 ± 2.4	3.6 ± 2.2	3.6 ± 2.0	3.7 ± 2.0	.035
*ROX index	5.3 ± 4.0	5.1 ± 3.8	4.7 ± 4.1	4.8 ± 4.0	.485
Prone position, No./total (%)†	9 (9.5)	24 (17.8)	193 (21.9)	226 (20.3)	.060
Prone position, duration(hour/session)	14.7 ± 7.5	17.1 ± 3.1	15.7 ± 8.0	15.8 ± 7.6	<.001
Total duration (day)	7.0 ± 6.9	10.3 ± 15.2	6.6 ± 7.0	7.0 ± 8.4	.002

BMI = body mass index, ECMO = extracorporeal membrane oxygenation, HFOT = high-flow oxygen therapy, ICU = intensive care unit, ROX = ratio of oxygen saturation, SOFA = sequential organ failure assessment.

* The worst values during 1st 24 h.

† Analysis of both MV and awakening prone position.

### 3.3. Comparison of clinical characteristics between waves according to level of respiratory care

In NIV or HFOT group, duration of NIV or HFOT (*P* = .035), length of hospital stay (*P* = .017), length of ICU stay (*P* = .001), and ICU mortality (*P* = .006) showed significant differences (Table 3-1). In invasive ventilation group, there were significant differences across the 3 groups in terms of the proportion of prone position (*P* = .003), length of ICU stay (*P* = .020), hospital mortality (*P* = .007), and ICU mortality (*P* = .008) (Table 3-2). In ECMO group, hospital mortality (*P* = .033) and ICU mortality (*P* = .041) showed significant differences across the 3 groups (Table 3-3). When collectively analyzing the mortality of the 3 groups, there was a significant difference in ICU mortality by wave in all 3 groups, but only in groups 2 and 3 for hospital mortality.

**Table 3-1:** Comparison of clinical characteristics between periods according to level of respiratory care (NIV or HFOT).

Clinical outcome	Noninvasive ventilation or HFOT			Overall (n = 921)	*P*-value
	Period 1 (n = 70)	Period 2 (n = 106)	Period 3 (n = 745)		
Clinical outcome					
Duration of noninvasive ventilation or HFOT	3.7 ± 4.3	5.9 ± 6.0	6.1 ± 7.3	5.9 ± 7.0	.035
ROX index	5.9 ± 3.7	5.6 ± 3.7	5.3 ± 3.9	5.4 ± 3.9	.386
Proportion of prone position, No (%)	5 (7.1)	15 (14.2)	146 (19.6)	166 (18.0)	.084
Prone position, duration(hour/session)	17.8 ± 6.6	17.4 ± 3.8	16.9 ± 7.3	17.0 ± 6.9	.936
Prone position, Total duration (day)	6.2 ± 3.0	6.9 ± 8.9	4.7 ± 4.6	5.0 ± 5.2	.247
Length of Hospital	33.0 ± 21.3	36.8 ± 35.7	28.3 ± 30.3	29.6 ± 30.5	.017
Length of ICU	25.7 ± 20.0	24.3 ± 22.7	18.8 ± 18.7	19.9 ± 19.4	.001
Mortality of Hospital	14 (20.0)	20 (18.9)	176 (23.6)	210 (22.8)	.465
Mortality of ICU	10 (14.3)	14 (13.2)	157 (21.1)	181 (19.7)	.006
Clinical frailty scale at discharge	2.9 ± 1.6	3.0 ± 1.4	3.2 ± 1.8	3.1 ± 1.8	.197

HFOT = high-flow oxygen therapy, ICU = intensive care unit, NIV = noninvasive ventilation, ROX = ratio of oxygen saturation.

**Table 3-2:** Comparison of clinical characteristics between periods according to level of respiratory care (IMV).

Clinical outcome	Invasive mechanical ventilation			Overall (n = 621)	*P*-value
	Period 1 (n = 60)	Period 2 (n = 80)	Period 3 (n = 481)		
Clinical outcome					
Duration of invasive ventilation	21.0 ± 19.8	25.1 ± 21.8	24.1 ± 24.7	23.9 ± 23.9	.573
ROX index	4.5 ± 4.1	3.8 ± 3.3	3.6 ± 3.2	3.7 ± 3.3	.214
Proportion of prone position, No (%)	8 (13.3)	24 (30.0)	169 (35.1)	201 (32.4)	.003
Prone position, duration (hour/session)	15.5 ± 7.6	17.1 ± 3.1	17.4 ± 6.9	17.3 ± 6.6	.730
Prone position, Total duration (day)	4.5 ± 3.4	7.0 ± 7.4	5.1 ± 4.7	5.3 ± 5.0	.224
Length of Hospital	48.5 ± 45.9	49.8 ± 45.5	40.5 ± 36.1	42.5 ± 38.6	.063
Length of ICU	38.4 ± 46.0	30.4 ± 25.4	28.2 ± 23.7	29.5 ± 27.0	.020
Mortality of Hospital	13 (21.7)	28 (35.0)	202 (42.0)	243 (39.1)	.007
Mortality of ICU	10 (16.7)	24 (30.0)	189 (39.3)	223 (35.9)	.008
Clinical frailty scale at discharge	2.8 ± 1.4	3.1 ± 1.4	3.2 ± 1.7	3.2 ± 1.7	.207

ICU = intensive care unit, IMV = invasive mechanical ventilation, ROX = ratio of oxygen saturation.

**Table 3-3:** Comparison of clinical characteristics between periods according to level of respiratory care (ECMO).

Clinical outcome	ECMO			Overall (n = 128)	*P*-value
	Period 1 (n = 13)	Period 2 (n = 15)	Period 3 (n = 100)		
Clinical outcome					
Duration of ECMO	26.7 ± 34.4	29.2 ± 27.2	28.2 ± 21.6	28.1 ± 23.7	.962
ROX index	3.5 ± 3.3	1.8 ± 3.0	3.3 ± 2.5	3.1 ± 2.7	.136
Proportion of prone position, No (%)	2 (15.4)	4 (26.7)	32 (32.0)	38 (29.7)	.450
Prone position, duration(hour/session)	6.0 ± 8.5	17.3 ± 1.2	15.2 ± 6.9	14.8 ± 7.0	.159
Prone position, Total duration (day)	1.5 ± 0.7	5.8 ± 2.2	5.1 ± 4.2	5.0 ± 3.9	.428
Length of Hospital	84.4 ± 82.8	53.4 ± 43.5	58.7 ± 47.0	60.7 ± 51.5	.202
Length of ICU	69.5 ± 87.0	42.8 ± 35.5	43.9 ± 29.5	46.3 ± 39.9	.087
Mortality of Hospital	3 (23.1)	9 (60.0)	61 (61.0)	73 (57.0)	.033
Mortality of ICU	3 (23.1)	8 (53.3)	60 (60.0)	71 (55.5)	.041
Clinical frailty scale at discharge	2.5 ± 1.2	2.5 ± 1.2	2.5 ± 1.4	2.5 ± 1.4	.989

ECMO = extracorporeal membrane oxygenation, ICU = intensive care unit, ROX = ratio of oxygen saturation.

### 3.4. Clinical predictors for hospital mortality in overall population

Multivariate Cox regression analysis indicated that age, initial SOFA, time wave, solid malignant disease, and ECMO use were significantly associated with mortality (age: Hazard ratio (HR) = 1.036, 95% CI = 1.022–1.050, *P* < .001; initial SOFA: HR = 1.167, 95% CI = 1.096–1.242, *P* < .001; time wave: HR = 1.528, 95% CI = 1.191–1.960, *P* = .001; solid malignant disease: HR = 1.678, 95% CI = 1.128–2.496, *P* = .011; ECOM use: HR = 1.765, 95% CI = 1.228–2.537, *P* = .002) (Table 4).

**Table 4:** Cox regression according to mortality.

	Univariate HR (CI)	*P*-values	Multivariate HR (CI)	*P*-values
Age	1.033 (1.022–1.043)	<.001	1.036 (1.022–1.050)	<.001
Sex	1.140 (0.900–1.444)	.278		
BMI	0.986 (0.961–1.013)	.312		
Smoking Hx.	0.829 (0.644–1.065)	.143		
Clinical frailty scale before admission	1.190 (1.118–1.266)	<.001		
Initial SOFA	1.198 (1.135–1.265)	<.001	1.167 (1.096–1.242)	<.001
Time period	1.457 (1.184–1.792)	<.001	1.528 (1.191–1.960)	.001
Hypertension	1.142 (0.903–1.443)	.267		
Coronary vascular disease	1.500 (1.099–2.046)	.011		
Pulmonary disease	1.471 (1.045–2.071)	.027		
Chronic neurologic disease	1.422 (1.065–1.897)	.017		
Chronic renal disease	1.510 (1.063–2.144)	.021		
Solid malignant disease	1.922 (1.366–2.704)	<.001	1.678 (1.128–2.496)	.011
NIV or HFOT use	0.893 (0.690–1.155)	.390		
IMV use	1.191 (0.869–1.632)	.277		
ECMO use	1.046 (0.911–1.202)	.522	1.765 (1.228–2.537)	.002

ECMO = extracorporeal membrane oxygenation, HFOT = high-flow oxygen therapy, HR = hazard ratio, ICU = intensive care unit, IMV = invasive mechanical ventilation, NIV = noninvasive ventilation, ROX = ratio of oxygen saturation, SOFA = sequential organ failure assessment.

**Figure 2.**
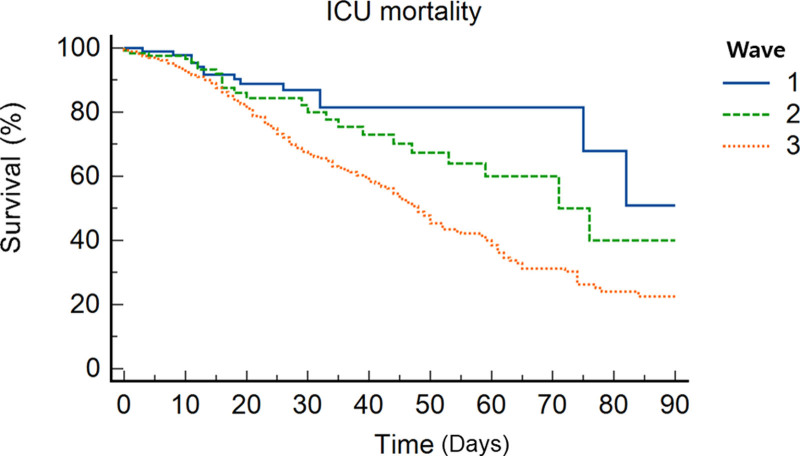
Kaplan–Meier analysis for ICU mortality according to the waves.

Kaplan–Meier curve revealed that time wave was associated with ICU mortality rate (χ^2^ = 9.683, *P* = .008, generalized Wilcoxon) (Fig. 2).

## 4. Discussion

During COVID-19, domestic healthcare resources showed sharp differences in utilization between regions and individuals.

In response to the COVID-19 pandemic, medical resources such as hospital beds were focused on treating patients with infectious diseases, and changes were made to the entire healthcare system, including the work of public health centers and medical services at hospitals. This has led to increased inequalities in access to healthcare for non-coronavirus patients and increased inequalities in healthcare services between regions.

In this study, the overall hospital mortality of patients with critical ill COVID-19 was 26.4%. The hospital mortality rates increased progressively from wave 1, wave 2, and wave 3, with the highest mortality rates occurring in wave 3. In this wave, the number of cases treated with ECMO increased, and the outcome of ECMO amounted to 61%, which was worse than that in wave 1 (23.1%). Age, clinical frailty scale before admission, initial SOFA, wave 3, and comorbidity were significantly associated with mortality.

The outcome of critical ill COVID-19 varied according to the country, medical resources, locoregional surge of outbreak, and demographic factors, ranging from 10 to 60%.^[13–17]^ Overall, the outcome of critical ill COVID-19 in this study was consistent with those in previous studies from other countries. Even though the national outcome of COVID-19 infection was better than that in other countries,^[8]^ the outcome of severe cases was not very different. To address this phenomenon, we analyzed the ICU data from each wave of early pandemic between Feb. 2020 and Aug. 2021.

All the 3 time waves showed no difference in hospital mortality, although there was a significant difference in ICU mortality, with the highest mortality rate in wave 3. Length of hospital stay and length of ICU stay were found to be the shortest in wave 3 compared to those in waves 1 and 2 (Table 2). Considering the attenuated toxicity and vaccination in wave 3, the worse hospital mortality could be associated with the surge of critical ill COVID-19 cases and overloading of ICU services.

We compared the patient’s characteristics, treatment pattern, and clinical outcome across the 3 time waves. While there was no significant difference in baseline clinical outcomes and disease severity between waves 1 and 2, a notable difference was that patients were treated with NIV or HFOT more frequently in wave 3. In wave 3, the number of days that patients received noninvasive ventilator was significantly higher than that in waves 1 and 2. While MV was most commonly used in waves 1 and 2 (wave 1: 49.4%, wave 2: 48.1%), a decrease in MV use (43.1%) and a significant increase in NIV or HFOT use (45.5%) were identified in wave 3. Use of high-flow nasal cannulation therapy raised concerns in the early days of the pandemic due to the risk of infection transmission to healthcare workers.^[18,19]^ However, due to the shortage of ventilators or locoregional surge of outbreaks, high-flow nasal cannulation therapy delivers a high percentage of humidified oxygen during treatment. It was more widely applied and became more popular due to its effectiveness in improving ventilation efficiency and breathing patterns, high tolerability, ease of use, and application outside of the ICU.^[18,20–22]^ The same trend was seen in Korea, and use of NIV or HFOT instead of conventional invasive respiratory support devices increased for patients with critical ill COVID-19 pneumonia.

The notable aspect of ICU resource usage over time is placing the patient in prone position. The frequency of application of prone position ventilation increased gradationally from wave 1, wave 2, to wave 3. In particular, the frequency of prone position application increased almost twice as much in wave 1 from wave 2 and continued until wave 3. In both waves 2 and 3, the ventilator group applied the prone position more than the ECMO and NIV or HFOT groups did. The increase in frequency of prone position ventilation and NIV or HFOT use might reflect the overburdening of ICU system. Shortage of dedicated ICU beds might induce more frequent use of NIV or HFOT and prone position, thereby minimizing the burden on ICU.

The risk factors identified in this study were consistent with those of previous studies.^[15,23–29]^ Age was the strongest risk factor among all demographic factors associated with mortality. The majority of deaths were attributed to age being above 65 years and solid malignant disease as comorbidity.

As we move from waves 1, 2, to 3, the length of ICU stay decreases. It is important to note that shorter ICU stays and higher ICU mortality are contrasting outcomes. Policy changes, such as the removal of ICU isolation, may have influenced these results. Unlike the original, the policy was changed to cover ICU stays up to 20 days after symptom onset and to allow patients with underlying respiratory conditions or stable care such as ventilators to be deconfined.

This study has several limitations; due to limited medical resources, dedicated intensive care beds might have been composed of mixed structures with general isolation room for ward patients and intensive care unit. Intensive care can be supplied in a distorted way. In addition, patients who died before admission to the dedicated intensive care bed were not included in the data. Another issue is that the case report form was modified due to the dedicated intensive care bed being different from the original ICU setting. Therefore, it lacks some important clinical ICU variables, such as disease severity, details of ventilator parameters, and nursing protocol. However, the current study has great significance, since it presents nationwide data regarding the details of clinical practice in a dedicated intensive care bed in Korea.

## 5. Conclusion

This study reported the largest cohort of patients with critical ill COVID-19 in Korea, presenting the details of ICU care during the early pandemic wave. The significance of this study is that it compiled and analyzed national data on trends and changes in critical care in the country.

The results suggest that medical treatment such as prone position and HFNO that were not previously in popular use may benefit patients during times of medical overload. In the event of an outbreak of a novel infection, the data may provide important experience to respond before its pathophysiology is fully revealed. Additionally, the data suggested an underpinning dataset that may contribute to the government’s policy development for any potential threat of novel pandemic infection in terms of intensive care system in future.

## Author contributions

**Conceptualization:** Taehwa Kim, Jeong Su Kim, Min Wook So, Hye Ju Yeo, Jin Ho Jang, Onyu Park, Woo Hyun Cho.

**Data curation:** Taehwa Kim, Min Wook So, Hye Ju Yeo, Jin Ho Jang, Onyu Park.

**Formal analysis:** Taehwa Kim.

**Validation:** Taehwa Kim, Woo Hyun Cho.

**Writing – original draft:** Taehwa Kim, Jeong Su Kim.

**Writing – review & editing:** Woo Hyun Cho.
